# Standards for reporting implementation studies (StaRI): enhancing reporting to improve care

**DOI:** 10.1038/s41533-017-0045-7

**Published:** 2017-06-26

**Authors:** Hilary Pinnock, Aziz Sheikh

**Affiliations:** Asthma UK Centre for Applied Research, Usher Institute of Population Health Sciences and Informatics, The University of Edinburgh, Edinburgh, EH* 9AG UK

The *npj Primary Care Respiratory Medicine* is extending its scope to include implementation science. Service delivery and organisation of healthcare has always been part of the journal’s remit, and many of the published papers already reflect the challenges of implementing evidence-based care,^1–11^ so this change is making explicit a long-held interest of the journal, its authors and readers. The International Primary Care Respiratory Group (IPCRG) in its Research Needs Statement prioritised real-life studies evaluating ‘overall management strategies’ over efficacy trials of specific drugs or treatments.^12, 13^ Similarly, the Primary Care Respiratory Society (PCRS-UK) includes ‘promoting and disseminating real life primary care research in respiratory conditions to support policy and education activities’ as one of its core activities.^14^ This extension of emphasis therefore resonates with both the parent primary care organisations.

**Fig. 1:**
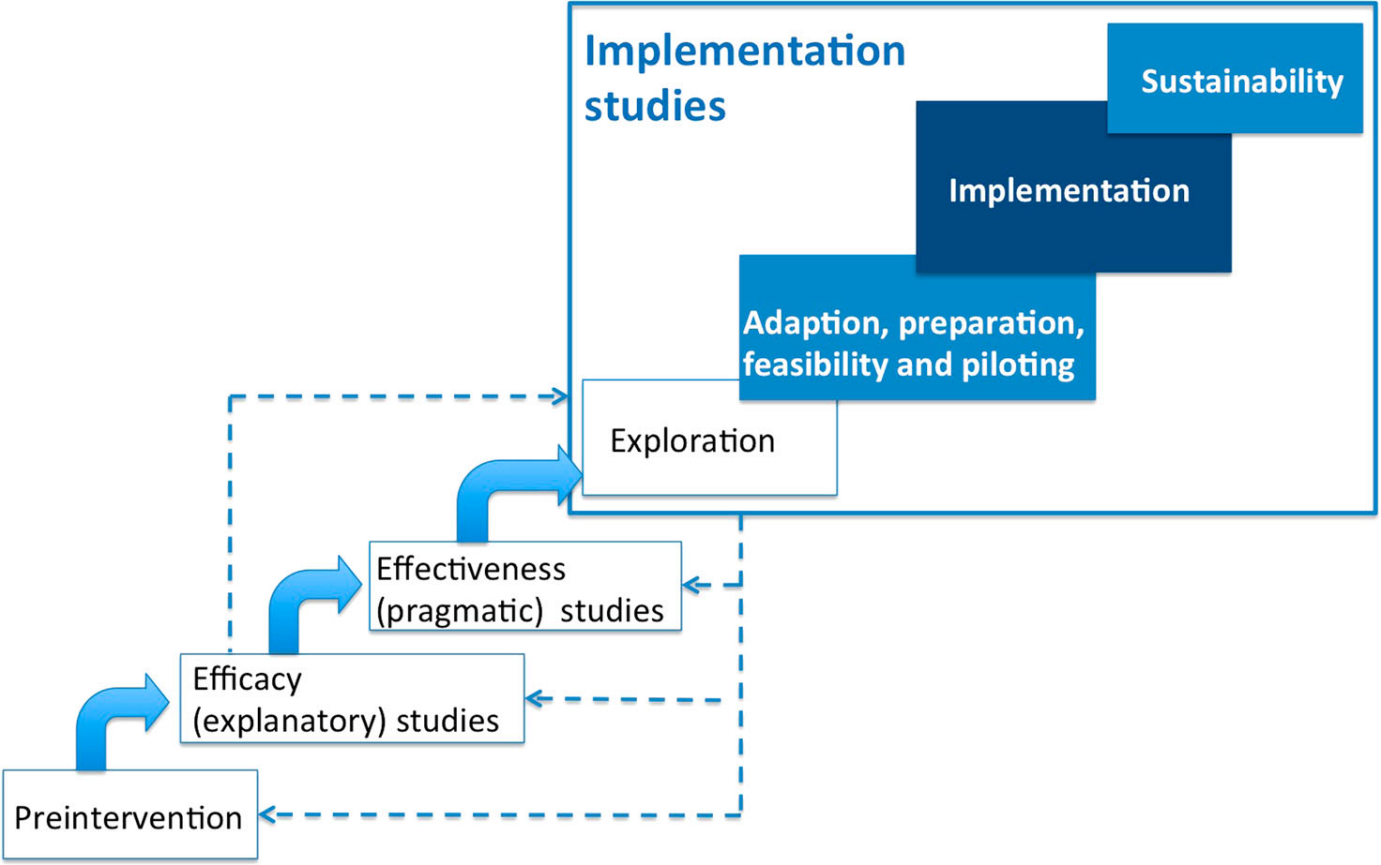
Positioning of implementation studies and the focus of StaRI reporting standards (reproduced with permission from Pinnock et al. [25]). StaRI is targeted on the reporting of interventional implementation studies (the *dark shaded box*) but will have resonance for studies in the pilot and sustainability phases

Implementation science is a relatively new discipline that aims to develop the evidence base on how to translate research findings into routine care.^15, 16^ This is a broad agenda which extends from preliminary work exploring the factors affecting implementation,^3, 10, 11^ developing implementation strategies,^1^ piloting the processes,^2^ testing the impact of implementation,^4, 8, 9^ and promoting scaling up and sustainability.^6^ This has been conceptualised as an additional cycle which takes an effective complex intervention,^17^ explores the existing context, and develops and evaluates strategies for embedding change in routine care.^18^ Other models have extended the spectrum of efficacy and effectiveness trials to encompass implementation science (see Fig. 1).^19^ The underlying tenet is the same: interventions may be effective in trials, but clinicians and healthcare organisations struggle to introduce them in the busy complex world of routine clinical practice. This may be a particular challenge in the context of primary care where a multitude of guidelines may be applicable to the diverse demands of front-line medical practice. A classic example is the poor implementation of self-management for asthma despite a long-standing and overwhelmingly positive evidence base.^20, 21^ Understanding the routines of primary care practice is the first step in developing a workable strategy that enables self-management to be embedded in practice.^11^

Existing methodologically-based reporting standards (e.g., CONSORT,^22^ STROBE,^23^ COREQ^24^) are not wholly appropriate for reporting implementation studies, though some of the methodological criteria will be useful. It is for this reason that we, together with colleagues, launched the Standards for Reporting Implementation studies (StaRI) initiative. *npj PCRM* will be encouraging authors of implementation studies to use StaRI, recently published in the BMJ,^25^ with an accompanying explanation and elaboration document.^26^ The checklist for authors to complete is freely available from http://www.bmj.com/content/bmj/suppl/2017/03/06/bmj.i6795.DC1/pinh034338.w2.pdf. Developed following an e-Delphi and an international consensus meeting, an underpinning theme of StaRI is the distinction between the implementation strategy (‘how the intervention was implemented’) and the evidence-based intervention that is being implemented. These dual strands run through the reporting standards with a requirement to consider the context, methods, outcomes and implications of both the implementation strategy and the intervention. The resultant clarity will, we hope, not only help authors of implementation papers, but will also be valuable for clinicians, heath service managers and researchers, planning initiatives to disseminate and implement guidelines and improve the quality of care.

In efficacy trials, the aim is to demonstrate that an intervention works when it is delivered as intended; ‘fidelity’ is thus prioritised and there will be little or no room for clinical or organisational manoeuvre. In contrast, in implementation studies, fidelity will be required to the core features of the intervention, but adaptation of the implementation strategy will be expected—indeed encouraged—to suit local organisational routines, demographic profiles and clinical context.^27^

Another feature of StaRI is the expectation that authors will detail how they believe the implementation strategy and intervention will work to improve health outcomes. This then leads logically to determining the key components of the implementation strategy and the process, implementation and health outcomes that should be measured. This is a discipline that will not only improve the quality of reporting implementation studies, but could also contribute to the development and evaluation of all healthcare initiatives.

One of the key challenges of using StaRI will be including the substantial descriptions of context, implementation strategy and intervention within the permitted word counts. We expect that authors will look for innovative ways to succinctly provide adequate detail (the Explanation and Elaboration document offers some suggestions^26^) and *npj PCRM* encourages on-line supplements for additional information. Publication of protocols, already the norm for randomised trials, will offer opportunity for publishing details of the implementation strategy and intervention.^5, 7^

Implementation studies resonate with primary care readers, but poor quality of reporting has made them difficult to identify, and their value is limited if descriptions are inadequate. By expecting adherence to the StaRI standards, *npj PCRM* hopes to contribute to efforts to improve the standard of reporting of implementation science thereby enhancing its value to researchers and clinicians, and ultimately raising the quality of care for people with respiratory conditions.
